# Trichogenic Silver-Based Nanoparticles for Suppression of Fungi Involved in Damping-Off of Cotton Seedlings

**DOI:** 10.3390/microorganisms10020344

**Published:** 2022-02-02

**Authors:** Shimaa A. Zaki, Salama A. Ouf, Kamel A. Abd-Elsalam, Amal A. Asran, Mohamed M. Hassan, Anu Kalia, Fawziah M. Albarakaty

**Affiliations:** 1 Botany and Microbiology Department, Faculty of Science, Cairo University, Giza 12613, Egypt; shim.shimshim@yahoo.com (S.A.Z.); salama@sci.cu.edu.eg (S.A.O.); 2 Plant Pathology Research Institute, Agricultural Research Centre, Giza 12619, Egypt; kamelabdelsalam@gmail.com (K.A.A.-E.); asran.amal@gmail.com (A.A.A.); 3 Department of Biology, College of Science, Taif University, Taif 21944, Saudi Arabia; m.khyate@tu.edu.sa; 4 Electron Microscopy and Nanoscience Laboratory, Department of Soil Science, College of Agriculture, Punjab Agricultural University, Ludhiana 141004, Punjab, India; kaliaanu@pau.edu; 5 Department of Biology, Faculty of Applied Science, Umm Al-Qura University, Makkah Al Mukarramah 21955, Saudi Arabia

**Keywords:** microbial synthesis, silver nanoparticles, antifungal activity, Egyptian cotton, soil-borne pathogenic fungi

## Abstract

Mycogenic silver nanoparticles (AgNPs) produced by some biocontrol agents have shown the ability to inhibit the growth of numerous plant pathogenic fungi, which may be a unique method of disease management. This study describes the extracellular production of AgNPs by *Trichoderma harzianum*. The size, shape, charge, and composition of the AgNPs were subsequently studied by UV-visible spectroscopy, DLS, zeta potential, TEM, SEM, and EDX, among other methods. The AgNPs had sizes ranging from 6 to 15 nm. The antifungal activities of bio-synthesized AgNPs and two commercial fungicides (Moncut and Maxim XL) were tested against three soil-borne diseases (*Fusarium fujikuroi, Rhizoctonia solani*, and *Macrophomina phaseolina*). Cotton seedling illnesses were significantly reduced under greenhouse settings after significant in vitro antifungal activity was documented for the control of plant pathogenic fungi. The use of biocontrol agents such as *T. harzianum*, for example, may be a safe strategy for synthesizing AgNPs and using them to combat fungus in Egyptian cotton.

## 1. Introduction

Cotton (*Gossypium barbadense* L.), which belongs to the family Malvaceae, is considered one of Egypt’s main cash crops. Cotton seedling diseases occur due to infection caused by a complex of soil-borne organisms, including *Rhizoctonia solani* Kühn (Teleomorph: one of the most primordial Basidiomycetes is Thanatephorus cucumeris (Frank) Donk). R. solani can be found in practically all cultivated fields in vegetative form [1]. In the preponderance of Egypt’s cotton-growing zones, *R. solani* was discovered to be the most important cause of cotton damping-off. A pathogenicity assay of *R. solani* belonging to different Anastomosis groups (AGs), i.e., Ag2-2, AG-4, AG-5, and AG-7, under greenhouse conditions revealed that most of the virulent isolates caused pre-emergence damping-off [2,3]. All of these isolates were extremely pathogenic, causing 100 % death in the pre-emergence stage [2,3,4,5]. *Fusarium* species are often isolated from diseased cotton roots and are recorded as a common pathogen of cotton seedling roots [6,7,8]. *Macrophomina phaseolina* (Tassi) Goid is a seed-borne and soil-borne disease and exhibits a wide host range that causes charcoal rot in cotton. Although early *M. phaseolina* infections in cotton occur in the seedling stage, they often remain until plant maturity [9]. When *M. phaseolina* penetrates cotton roots or stems, internal tissues are colonized quickly, and the plant dies. Dry rot is observed on diseased portions, with the occurrence of black sclerotia scattered throughout the wood and softer tissue [1].

The fungus-mediated control of pathogenic fungi is an environment friendly, cost-effective, and biocompatible approach to managing the phytopathogens [10]. The use of fungal-mediated green chemistry techniques for the synthesis of NPs can improve yield potential while lowering the input costs [11]. As a result of their high processing capacity, substantial surface area recovery, and excellent mycelial growth, fungi are an efficient and easy biosynthetic scaling agents [12]. Mycogenic nanoparticles (NPs) synthesized by beneficial fungi is a potential environmentally acceptable approach for large-scale manufacturing of various nanoparticles. Various fungal species have been used for the biosynthesis of silver nanoparticles, including *Trichoderma koningii, Aspergillus flavus*, *Fusarium oxysporum*, *Penicillium citrinum*, and *P. fellutanum* [13]. One of the most significant plant species in agriculture is *T. harzianum*, which serves as a biological control agent against phytopathogens. Hyaloid development and the release of hydrolytic enzymes breakdown the fungus’s cell wall as its primary mode of action. Enzymes that are critical to mycoparasitism include chitinases, N-acetyl-β-D-glucosamine deacetyltransferase, and proteases [14,15,16]. In the fields of biotechnology and nanotechnology, *T. harzianum* has been extensively studied, offering new possibilities for the development of new products and uses, due to its excellent qualities for the management of phytopathogens and the simplicity with which it may be handled [17,18].

*T. harzianum* is employed for stabilization in the green manufacture of biogenic silver nanoparticles. Secondary metabolites released by biocontrol agents (*T. harzianum*) operate as capping and reducing agents, which can help to produce uniformly sized green and sustainable nanomaterials [18,19,20]. *T.* *harzianum* cell filtrate has been used to produce biogenic AgNPs in a simple, green, and eco-friendly way, without the need for any toxic reducing, capping, or dispersion agents [21]. The generated nanoparticles characterized using physicochemical methods exhibited face-centered cubic symmetry [21,22]. Because this fungus has a variety of qualities that aid with agricultural commodities, *Trichoderma*-based products contribute to more than half of the overall worldwide biopesticide industry employed for improving plant growth, nutrient use efficiency, and physiological response to both biotic and abiotic stresses [23]. The use of green AgNPs as an antifungal agent is seen as an environmentally benign resource, an alternative to fungicides, and a cost-effective method [24,25]. Based on updated literature, the primary goals of this investigation were to: (1) determine the ability of a cell-free extract from a *T. harzianum* natural biocontrol strain to act as a reducing agent and stabilizer in the production of AgNPs; (2) undertake physicochemical characterization to determine the structure, size, and morphology of the generated AgNPs; and (3) test the antifungal activity of AgNPs in vitro and in vivo against three fungal pathogens, namely, *R. solani, F. fujikuroi,* and *M. phaseolina*. 

## 2. Materials and Methods

### 2.1. Trichoderma Isolates 

Soil samples were taken from healthy cotton root rhizosphere soils and stored at 4 °C until used. Each soil sample was diluted five times in sterile distilled water before being applied to the surface of potato dextrose agar (PDA) (M/s HiMedia Pvt. Ltd., Mumbai, Maharashtra, India) with 0.5% of the diluted sample [26]. The single spore isolation technique was used to isolate putative *Trichoderma* colonies [27]. Petri plates inoculated with fungal strains were incubated at 28 ± 2 °C for 4 days. At the Assiut University Mycology Center (AUMC) in Assuit, Egypt, morphological approaches were used to identify *Trichoderma* species. Eppendorf tubes with sterile deionized water were used to maintain the isolates. The fungus was re-grown at 25 °C for 5 days in the dark on the freshly prepared PDA.

### 2.2. Preparation of Fungal Filtrate and Tricoh-Synthesis of Silver Nanoparticles

To prepare biomass for biosynthetic studies, *Trichoderma* isolates were inoculated in 250 mL conical flasks containing modified TLS liquid broth in addition to yeast extract (0.6 g/L). *T. harzianum* biomass (20 g) was mixed with 100 mL sterile deionized water in a flask. To purify *T. harzianum* filtrate, the biomass was agitated and filtered with Whatman filter paper no. 1 after incubation [28].

For three days, 50 g of fungal cell filtrate cultures were agitated at 150 rpm with 50 mL of 1 mM AgNO_3_ solution. The fungal biomass filtrate without the silver nitrate solution served as a positive control, whereas the silver nitrate solution without the cell-free filtrate served as a negative control. Centrifugation (12,000 rpm for 20 min) was used to separate the nanoparticles, which were then washed twice with double distilled water and lyophilized for 24 h. Silver nanoparticles were generated by converting silver ions to metallic silver. The NPs were then kept in polypropylene tubes in the dark at room temperature. The generated AgNPs were subjected to several analytical methods for physicochemical characterization [19].

### 2.3. AgNPs Characterization

#### 2.3.1. UV-Vis Spectrophotometer Analysis

The essential approach and the easiest way to establish the synthesis of nanoparticles in colloidal solution is via UV-Vis spectroscopy (T80 UV-Vis spectrophotometer, PG Instruments Ltd, Leicestershire, LE17 5BH, United Kingdom). After 2, 4, and 7 days of incubation, periodic sampling of 1 mL of the solution was performed, and the samples were scanned for a wavelength range of 0 to 1100 nm on a UV-Vis spectrometer. The visual shift in the color of the solution from yellow to brown was used to observe the reduction in metal ions. The distilled water was used as a blank to set the reference. 

#### 2.3.2. Transmission Electron Microscopy (TEM)

TEM was performed to ascertain the shape and measure the size of the biogenic AgNPs. The sample was made by depositing an aliquot of an aqueous AgNPs suspension onto a carbon-coated copper grid and allowing the contents to air dry in a vacuum desiccator. The dried grids were viewed in a TEM (Leo 912 AB OMEGA, Carl Zeiss, Oberkochen, Germany) operating at an accelerating voltage of 80 kV. The particle size distribution of the biogenic AgNPs was obtained through image analysis of the acquired TEM micrographs by Image J software (version 1.45, NIH, USA).

#### 2.3.3. Dynamic Light Scattering and Zeta Potential assays

The particle size distribution of the biosynthesized AgNPs was also investigated through dynamic light scattering spectroscopy (Malvern Instruments, Southborough, MA, USA). In the data processing mode, the multi-modal resolution was set to high. The synthesized AgNPs solution was diluted (20, 40, 60, 80, and 100%), and the diluted sample was either filtered or left unfiltered using a 0.22-μm syringe-driven filter unit. All measurements were taken in triplicate at 25 °C with a 1-min temperature equilibration delay. 

To determine the stability and surface charge of AgNPs obtained from *T. harzianum*, zeta potential analysis was undertaken and the average hydrodynamic diameter of the nanoparticles were also obtained from the measurements (polarity, viscosity, conductivity, charge, pH, etc.). The zeta potential of aqueous AgNPs suspension was determined on a Mansetting nano (Malvern Instruments, Southborough, MA, USA) zeta sizer equipped with Zetatrac and using Microtrac FLEX Operating Software. The sample was produced by dissolving AgNP powder in deionized water and sonicating it for 10 min in a bath sonicator (Wise WiseClean WUC A22H, Witeg Labortechnik GmbH, Wertheim, Germany).

#### 2.3.4. Scanning Electron Microscope (SEM)

SEM investigation was undertaken to identify the size of agglomerates of the biogenic AgNPs. Initially, the dried specimen was suspended in ethanol. On a carbon-coated copper grid, a very thin coating of the sample was dropped, and a thin film of the sample was generated. The sample was then examined on a scanning electron microscope (Vega-3 SBU, Tescan, 623 00 Brno-Kohoutovice, Czech Republic) operated at a 20 kV accelerating voltage.

#### 2.3.5. Energy Dispersive X-ray (EDX) 

The EDX analysis was performed on the generated AgNPs on an EDS system (JEOL JEM-1230, Tokyo, Japan) attached to a scanning electron microscope to evaluate the presence of elemental Ag. The gold coating was performed using a gold sputtering unit (Polaron E5100, Quorum Technologies Ltd., Laughton, East Sussex England) after the dried AgNPs were mounted on the copper mesh.

### 2.4. In Vitro Antifungal Activity of AgNPs

The in vitro antifungal potential of biosynthesized AgNPs was evaluated through the agar well diffusion method. *R. solani* (RS9), *Fusarium fujikuroi* (FF10), and *M. phaseolina* isolate (MP4) were cultured for 7 days on a PDA medium at 35 °C. A cork borer was used to cut 5 mm discs of fungal inoculum and inoculate a 9 cm diameter Petri dish, which was incubated at 27 °C for 5−7 days, and the freshly generated PDA containing varying concentrations of generated AgNPs (20, 40, and 100 µg/mL) was allowed to harden; three replicates were undertaken for each concentration. Controls were nanoparticle-free PDA plates cultured under the same conditions. The fungal mycelia radial growth diameter was measured after incubation.

### 2.5. Antifungal Activity under Greenhouse Conditions

The experiments were carried out in pots using the complete randomized block design (CRBD) according to the method developed by the authors [18]. The effects of the synthesized AgNPs against three soil-borne pathogens on the cotton types Giza 90 and Giza 94 were tested using the RS9, FF10, and MP4 pathogens. Pots of autoclaved soil were infected with two-week-old cultures of the fungal-sorghum *R. solani* (RS9), *Fusarium fujikuroi* (FF10), and *M. phaseolina* (MP4) at rates of 1, 50, and 50 g/kg soil, for each pathogen, respectively, to determine the effectiveness of the treatments (Table 1). For two minutes, NaOCl was used to sterilize cotton seeds from the Giza 90 and Giza 94 kinds before they were washed four times with sterile distilled water. The sterilized seeds were then immersed for 12 h under static conditions in AgNP suspensions at concentrations of 100 and 200 µg/mL. Moncut (2 g/kg seeds) and Maxim XL (2 mL/L) were added as a coating film for cotton seeds of Giza 90 and Giza 94. The infected soil was placed into 15 cm pots with 10 seeds per container. Only in the control condition, the sterilized sorghum grains were thoroughly combined with soil. Infected controls received 1 g/kg of *R. solani* soil (RS9) and 50 g/kg of *F. fujikuroi* (FF10) and *M. phaseolina* (MP4) with no treatment. The pots were randomly sprinkled on the greenhouse bench and repeated 3 times to obtain accurate data. There were 3 duplicates (pots) for each process. Plant height, dry weight, and survival rate of plants were calculated 45 days after sowing [19,29]. 

### 2.6. Statistical Analysis

The experimental design of laboratory and greenhouse experiments was a randomized complete block in a factorial arrangement with three replicates (blocks). The least significant difference (LSD) test was used to examine the mean differences at *p* ≤ 0.05. The MSTAT-C software was used to carry out the analysis of variance (ANOVA) of the data. 

## 3. Results

### 3.1. Characterization of Synthesized Silver Nanoparticles

#### 3.1.1. UV-Visible Spectral Analysis

**Figure 1:**
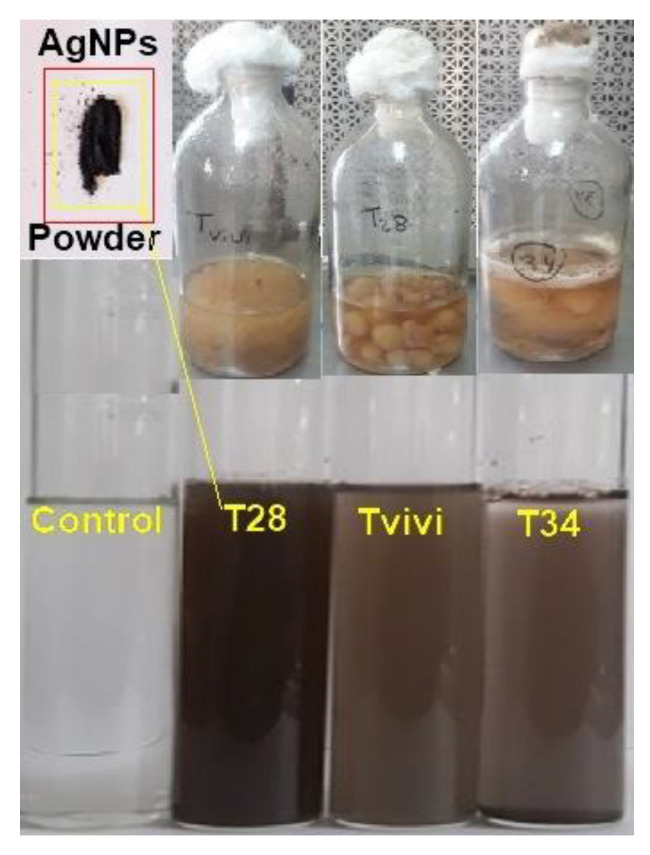
*Trichoderma* mycelium was produced within the liquid media of three isolates, namely, T28, Tvivi, and T34, in a 1 mM silver nitrate aqueous solution. The amount of brown hue in the sample shows the presence of silver nanoparticles (AgNPs).

A visual examination of the culture flasks revealed a color shift after the addition of AgNO_3_ to the mycelia free cell filtrate. After 5 min, the color of the AgNO_3_-containing mycelial free cell filtrate altered from colorless to light brown, followed by the emergence of dark brown (Figure 1). 

**Figure 2:**
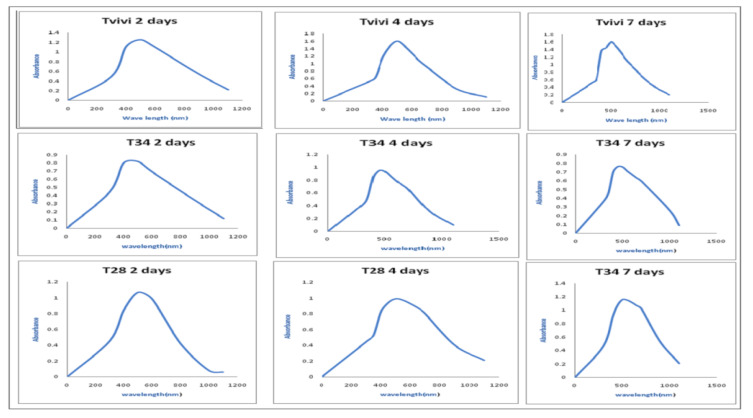
The UV-Vis spectrum of AgNPs generated by Tvivi, T34, and T28 was explored after 2, 4, and 7 days of synthesis.

The UV-Vis spectra of the mycelia-free cell filtrate incubated with silver nitrate salt of three *Trichoderma* isolates (Tvivi, T34, and T28) was obtained (Figure 2). The UV-Vis spectra of AgNPs derived from Tvivi, T34, and T28 revealed absorbance maxima of 500, 500, and 450 nm, respectively. AgNPs produced by Tvivi’s strain were chosen for further investigation.

#### 3.1.2. Dynamic Light Scattering (DLS) and Zeta Potential Analysis

**Figure 3:**
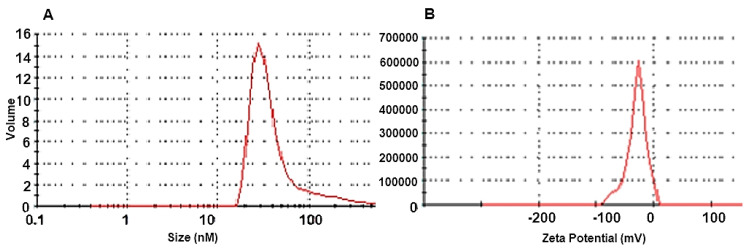
(**A**). Nanoparticle size distribution of silver nanoparticles (AgNPs) solution synthesized by Tvivi, (**B**) analysis of the zeta potential of synthesized AgNPs.

The particle size distribution of AgNPs solution as determined by DLS (Figure 3A) indicated an average particle size of 52.34 nm, PdI: 0.359, and a zeta potential of −25.1 mV (Figure 3B).

#### 3.1.3. Transmission Electron Microscope (TEM) Analysis

**Figure 4:**
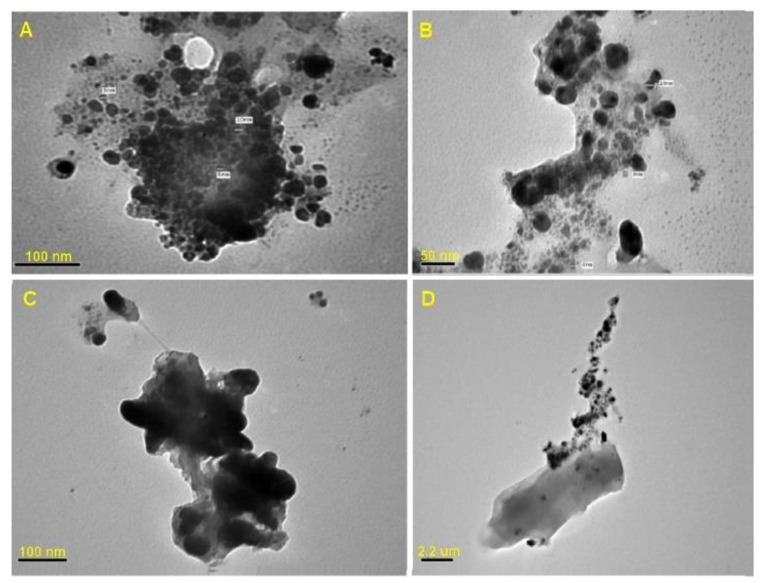
The TEM micrographs of the Tvivi produced silver nanoparticles (AgNPs) show the corresponding particle size distribution and shape. The electron micrographs were acquired at different magnifications **A** (×200 K), **B** (×360 K), **C** (×400 K), and **D** (×100 K).

The TEM images revealed spherical shaped AgNPs (Figure 4) with a particle size range of 6 to 15 nm, and the biogenic AgNPs were observed to be surrounded by a thin layer of organic material.

#### 3.1.4. Scanning Electron Microscope Analysis (SEM)

**Figure 5:**
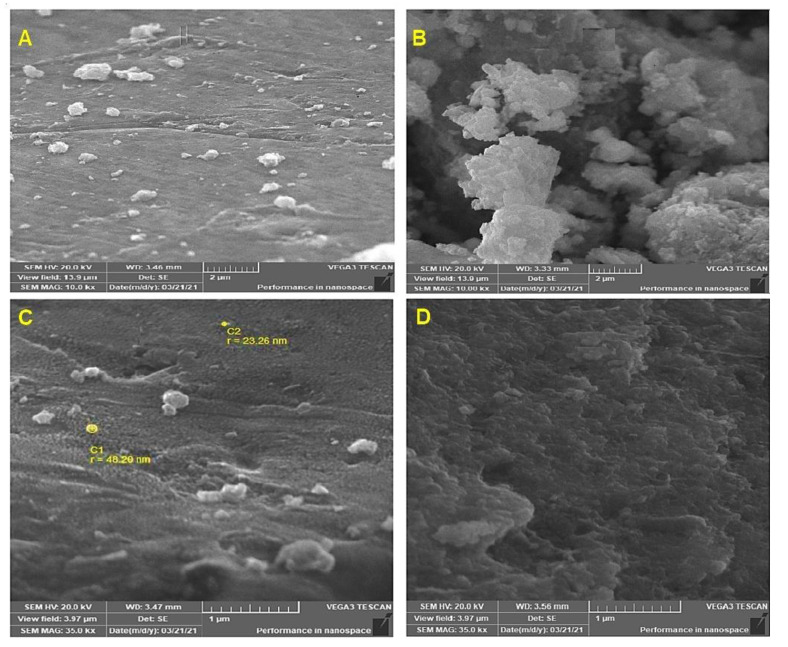
Scanning electron micrographs of the Tvivi synthesized silver nanoparticles (AgNPs) at 10.0 kX (**A**,**B**) and 35.0 kX (**C**,**D**) magnifications.

The SEM micrographs clearly showed nanoparticles with almost spherical shapes. Individual nanoparticles are grouped into clusters (Figure 5).

#### 3.1.5. Energy Dispersive X-ray Spectroscopy (EDX) Analysis

**Figure 6:**
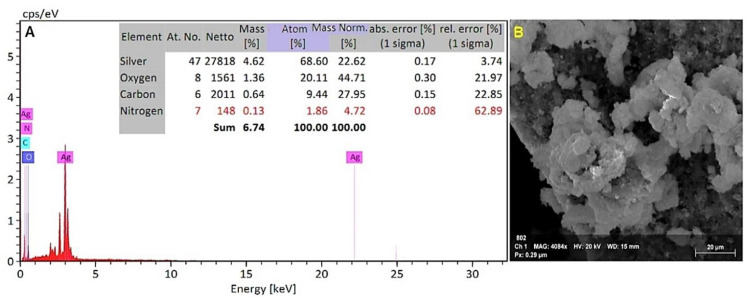
(**A**). The EDX spectrum and elemental analysis of the EDX spectrum of synthesized silver nanoparticles. (**B**). Screened area for the EDX spectrum of synthesized silver nanoparticles.

An EDX detector attached to a scanning electron microscope was used to determine the elemental composition of the powdered sample. The EDX investigation showed a strong signal for a silver element at around 3 keV (Figure 6A). The elemental analysis revealed atom (%) information as 68.60% silver, 20.11% oxygen, 9.44% carbon, and 1.86% nitrogen. 

#### 3.1.6. Antifungal Activity of AgNPs under In Vitro Conditions

The effects of the three concentrations of the AgNPs evaluated, and their interactions, were highly significant causes of variation (*p* = 0.00) for the fungal growth of all the studied fungi, FF10, RS9, and MP4, according to the ANOVA in Table 2.

**Figure 7:**
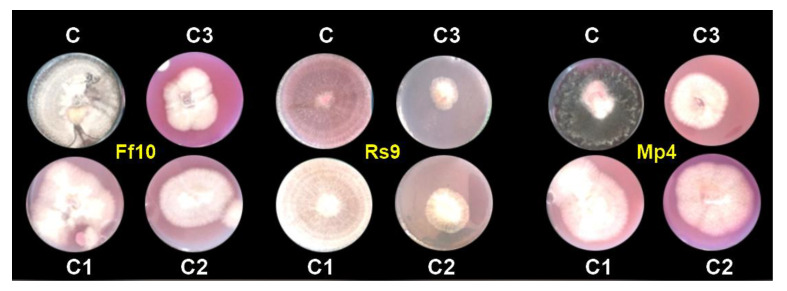
In vitro effect of AgNPs against damping-off pathogens caused by FF10, RS9, and MP4 isolates; (C) control, (C1) 20, (C2) 40 and (C3) 100 µg/mL after 7 days.

Because the concentration of AgNPs × fungus interaction had a significant influence on the radial growth of fungi, to analyze the radial growth averages of treatments within each fungus in Table 3, the LSD was calculated. These studies revealed that the variations in radial growth between concentrations and controls were not the same for each fungus, indicating that fungi reacted differently to concentrations. Thus, all AgNP concentrations were effective in decreasing the linear growth of all fungi compared to the control. In addition, the AgNP concentration of 100 µg/mL was the most effective in decreasing the linear growth, as it decreased linear growth to 4.000, 2.250, and 4.167 cm for FF10, RS9, and MP4, respectively, compared to the control (9 cm); see Figure 7. 

#### 3.1.7. Antifungal Activity of AgNPs under Greenhouse Pot Conditions

The number of surviving seedlings, plant height, and dry weight were improved when treatments were applied to infected soil (Table 4). Treatment was a highly significant cause of variation (*p* = 0.001) of all the examined variables, according to the ANOVA shown in Table 3. Only in the case of plant height was the fungus × treatment interaction a highly significant source of variance (*p* = 0.00). 

Because there was no fungus treatment interaction on survival, the general mean was used to compare treatment means (Table 5). Treatment with AgNPs (100 µg/mL) was the least effective treatment in controlling the condition, whereas other treatments were similarly beneficial. The difference between the examined fungi’s general means was non-significant. 

**Figure 8:**
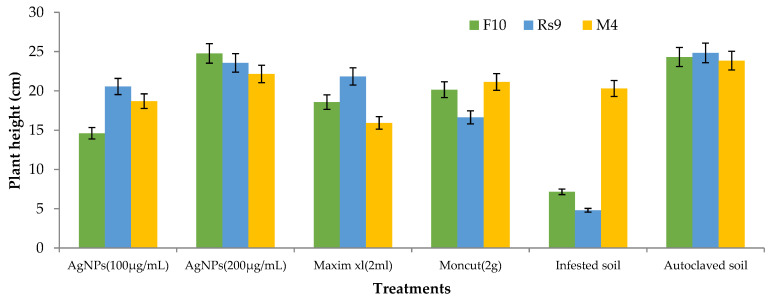
Effects of fungus, treatments, and their interactions on the plant height of Giza90 cotton seedlings cultivated in contaminated soil in a greenhouse. LSD (*p* ≤ 0.05) for fungus × treatment = 5.74.

**Figure 9:**
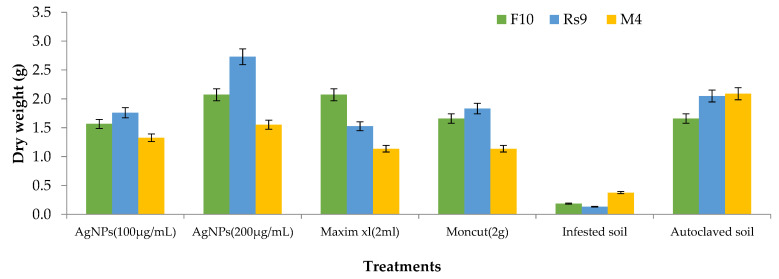
The effect of specific fungi, treatments, and their interactions on the dry weight of Giza 90 cotton seedlings grown in a greenhouse on infected soil. LSD for treatments = 0.43 (*p* ≤ 0.05). The LSD (*p* = 0.05) for fungus was non-significant.

Because the interaction between cultivar treatment and plant height was significant (Figure 8), the LSD value of interaction was used to compare treatment averages within each tested fungus. In the case of *F. fujikuroi*, AgNPs (200 µg/mL) and Moncut (2 g) demonstrated the greatest efficacy in disease control (FF10). In the instance of *R. solani*, all treatments were equally effective in controlling the disease (Rs10). In the case of *M. phaseolina*, all treatments were successful in managing the disease; however, treatment with Maxim xl (2 mL) was the least effective, whereas other treatments were similarly beneficial (MP4). Due to the lack of a fungus treatment interaction on dry weight (Figure 9), the general mean was utilized to compare treatment means. All treatments were equally successful in bringing the condition under control. The difference in general means of the investigated fungus was not statistically significant. 

ANOVA revealed that treatment was a very significant source of variation (*p* = 0.00) for all examined variables (Table 6). Fungus and the fungus × treatment interaction were not significant contributors to the variance in any of the variables evaluated. Because there was no fungal treatment interaction on survival, the overall mean was used to compare treatment means (Table 7). Although all treatments were successful in managing the condition, treatment with AgNPs (100 µg/mL) was the least effective, whereas the other treatments were all equally effective. The difference between the examined fungi’s overall means was not significant. 

**Figure 10:**
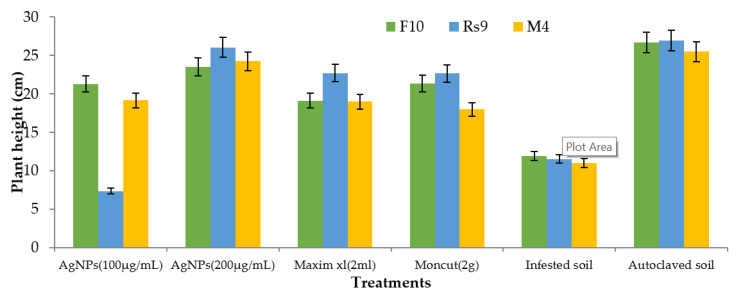
The effect of fungus, treatments, and interactions on the plant height of Giza 94 cotton seedlings cultivated on infected soil under greenhouse conditions. Treatment LSD (*p* ≤ 0.05) = 5.39. The LSD (*p* ≤ 0.05) for fungus was non-significant.

**Figure 11:**
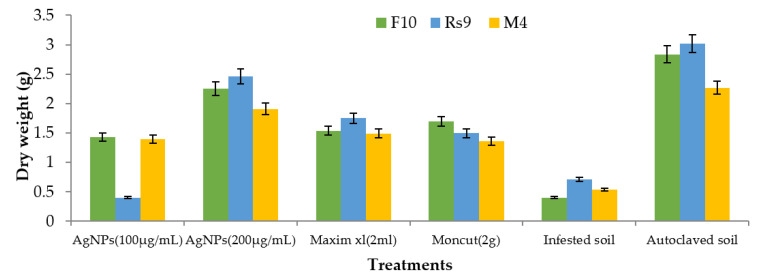
The effect of fungus, treatments, and interactions on the dry weight of Giza94 cotton seedlings cultivated on infected soil under greenhouse conditions is yet to be determined. LSD (*p* ≤ 0.05) for treatments = 0.55. The LSD (*p* ≤ 0.05) for fungus was non-significant.

**Figure 12:**
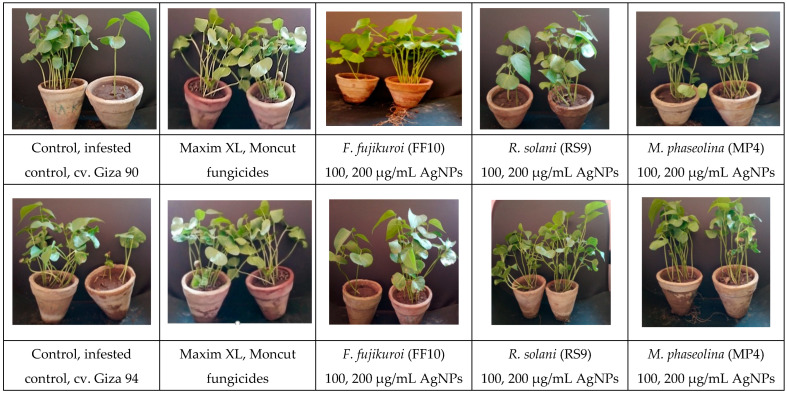
Uncoated cotton seeds sown in sterilized soil infested with three fungal pathogens, namely, *F. fujikuroi, R. solani*, and *M. phaseolina* as a negative control, uncoated cotton seeds sowed in sterilized soil as a positive control, treated with two fungicides (Maxim XL and Moncut) sowed in infested soil and coated with AgNPs (100, 200 µg/mL) in infested soil. The photo was obtained after 45 days of typical growth in a greenhouse.

Because there was no interaction between fungus and treatment on plant height and dry weight, the general mean was used to evaluate the means of the different treatments (Figure 10 and Figure 11). All treatments were effective in controlling the disease for plant height. However, the therapy with AgNPs (100 µg/mL) was the least effective, whereas the other treatments were all equally effective. In terms of dry weight, all treatments were efficient in managing the disease, apart from AgNPs (100 µg/mL) (Figure 12), which was ineffective, and AgNPs (200 µg/mL), which was the most effective treatment in terms of dry weight (2.208 g). There was no statistically significant difference in the general means of the fungi that were tested.

## 4. Discussion

*Trichoderma* is the most extensively used biocontrol agent, which exhibits characteristic features such as pathogen antagonism, stimulation of systemic resistance in the host, nutrition competition, and overall plant growth enhancement [21]. Biogenic AgNPs from *T. harzianum* extracellular filtrate have an advantage over physical and chemical processes that need high pressure, energy, and chemical precursors [19].

The ability of three *Trichoderma* species (Tvivi, T34, and T28) to generate stable AgNPs was examined. Filtrates from each fungal isolate were grown in the dark at 28 °C for 72 h with AgNO_3_ solution. After the incubation time, the color changed from pale yellow to dark brown. The emergence of the brown color indicated that AgNPs were forming in the media [28]. The UV-visible spectrum of biosynthesized silver nanoparticles utilizing (Tvivi, T34, and T28) displayed a peak at 500, 500, and 450 nm, respectively, on all tested days, matching silver nanoparticle plasmon absorbance. The findings were consistent with previously published reports on the UV absorbance peak of generated AgNPs [29,30,31].

The produced particles were monodispersive. The particle size estimated by TEM was smaller than the particle size reported by DLS measurements. This is because the hydrated capping agents, proteins, and solvation effects considerably increase the particle size obtained by DLS. The findings were in line with the expectations of some previous research reports [21,28,32,33].

In the present work, the zeta potential was found to be −25.1 mV. The negative value of the zeta potential indicates particle repulsion, which increases particle stability. The findings agreed with those of Ebrahimzadeh et al. [33], who obtained biosynthesized silver nanoparticles with a zeta potential of –19.6 mV. Moreover, Ahluwalia et al. [21] obtained a zeta potential of –17.19 mV. 

The morphology of silver nanoparticles produced was visualized through TEM analysis. Individual and aggregated monodispersed spherical silver nanoparticles with sizes ranging from 6 to 15 nm were observed. The NPs were coated with a thin layer of organic material within the aggregates, indicating that the NPs were stabilized by a capping agent [21,34]. Elamawi et al. [32] found that silver nanoparticles biosynthesized by *Trichoderma longibrachiatum* had monodispersed approximately spherical forms with varying diameters and a particle size distribution ranging from 1 to 25 nm. According to Nayak et al. [35], AgNPs biosynthesized by *Dillenia indica* extract were spherical, with the majority of them clustered and a few dispersed with varying sizes, as was described under TEM. 

SEM images showed AgNPs having a spherical form. Clusters of AgNPs were seen, which may be the result of nanoparticle aggregation during sample preparation. These factors may have had a role in the variance in particle size. The results corroborated those of Ahluwalia et al. [21], who reported that silver nanoparticles biosynthesized by *T. harzianum* exhibited spherical AgNPs with a range of diameters within the standard error of the mean. According to Tomah et al. [36], SEM micrographs revealed that the exterior surfaces of AgNPs generated from cell-free *T. virens* HZA14 filtrate were spherical with a size range of 5 to 50 nm. Hirpara et al. [24] demonstrated that silver nanoparticles biosynthesized by *T. interfusant* had spherical and pseudospherical forms with agglomeration traces and average size distribution of 59.66 ± 4.18 nm, which was under the standard error of the mean. 

The existence of AgNPs produced from the filtrate was verified by EDX analysis. This study revealed a high concentration of AgNPs (68.60 percent), followed by oxygen, carbon, and nitrogen, indicating that extracellular organic moieties from mycelial free cell filtrate were adsorbed on the nanoparticles’ surface [21]. Due to surface plasmon resonance, metallic AgNPs typically exhibit an optical absorption peak at 3 keV. The results corroborated those of Konappa et al. [20], who found the existence of AgNPs (58.75 percent) at 3 keV, followed by carbon, oxygen, and chlorine. According to Tomah et al. [36], EDS examination of AgNPs found that pure silver (28.85 percent) at 3 KeV was the second most abundant component element after oxygen, nitrogen, carbon, and sulfur. Additionally, Manikandaselvi et al. [37] revealed that significant signals were observed from the Ag atoms in the nanoparticles, in addition to signals from Al, carbon, and oxygen. These signals were most likely caused by X-ray emission from the fungus’s existing proteins.

In vitro, the antifungal activity of the produced AgNPs against *R. solani* (RS9), *F. fujikuroi* (FF10), and *M. phaseolina* (MP4) was determined. It was discovered that as the quantity of AgNPs increased, the diameter of the mycelium decreased. All concentrations of AgNPs were effective at reducing radial growth of all fungi when compared to the control, but AgNPs at 100 µg/mL was the most effective at reducing linear growth, as it reduced linear growth by 55.6, 75%, and 53.7% for the three pathogenic fungi FF10, RS9, and MP4, respectively.

Regardless of fungus, all treatments were successful in suppressing disease and improving life on both cotton cultivars. However, the treatment with AgNPs (100 µg/mL) was the least effective, whereas the other treatments were similarly beneficial. Commercial chemical fungicides (Maxim XL and Moncut) had the same impact as AgNPs (200 µg/mL). However, AgNPs were utilized at relatively low doses compared to other commercial chemical fungicides. Furthermore, it was discovered that using AgNPs at 200 µg/mL was more successful than using 100 µg/mL in suppressing disease and boosting survival, independent of fungus. 

Several papers [38,39,40,41,42] emphasize the potential approach of AgNPs for the synthesis of new antibacterial agents. Our findings, which agree with those of other researchers, demonstrate the feasibility of achieving a better inhibition rate against a wide spectrum of harmful bacteria, even at lower AgNP concentrations [43,44,45]. This may be due to AgNPs’ great propensity for attacking and adhering to fungal mycelium, destroying membrane integrity, and thereby suppressing the plant pathogen [46]. 

*M. phaseolina* fungal growth was suppressed by synthesized AgNPs with a zone of inhibition of 13 mm at 1000 µg/mL according to Vijayabharathi et al. [47]. AgNPs may disrupt the lipid bilayer, in addition to membrane permeability and electrical potential, resulting in ion and other chemical leakage and cell death [48]. The antifungal activity of biosynthesized AgNPs was tested against eight *Fusarium* species that cause cotton seedling damping-off. On Czapek Dox agar and potato dextrose agar plates, in vitro treatments with varying amounts of AgNPs were produced. From 25 to 200 ppm of AgNP interaction, fungal growth was significantly slowed [49]. Silver nanoparticles were tested for their ability to inhibit *R. solani*-induced cucumber damping-off. The fresh and dry weights of roots and shoots were considerably greater in treatments containing 30, 50, and 70 g/L of nanoparticles than in control plants. It was discovered that nanosilver harmed *R. solani* when compared to the control. 

The capacity of AgNPs to permeate the cytoplasmic membrane and cell wall, RNA and DNA damage, and cell respiration all contribute to their lethal effects [50]. The NADH co-enzyme, in addition to NADH-dependent enzymes such as nitrate reductase, are found in *Trichoderma* genus strains and are vital in the creation of both nanoparticles and cappings that provide higher stability [51,52]. *T. harzianum* generates enzymes and metabolites that are involved in breaking strong ionic connections between silver and nitrate ions for its own life, perhaps through the action of hydrolytic/nitrate reductase enzymes. The enzymatic activity of extracellular fungal secondary metabolites converts hazardous Ag^+^ ions into innocuous biosilver NPs. The existence of suitable functional groups in *T. harzianum*’s extracellular filtrate, which is more effective than that of other fungi and non-toxic to humans [53], provides the basis for silver bioreduction [22]. 

Secondary metabolites are secreted by *T. harzianum* and act as capping and reducing agents, aiding consistency and contributing to biological activity. Puerarin, genistein, isotalatizidine, and ginsenoside were identified using LC-MS/MS [20]. AgNPs can interact with the cell wall and membrane, resulting in (1) changes in permeability; (2) phosphate management disruption; (3) plasma membrane disintegration; (4) proton motive force failure; (5) ATP production suppression; (6) an impact on amino acids and enzymes that can result in bonding with amino acids and inhibition of enzyme activity due to attachment to the active core of the enzyme; (7) energy-use impediments that can lead to electron mobility in the respiratory chain and cytochrome inhibition; (8) an effect on DNA and RNA that can cause hydrogen bonding to break, nitrogen base synthesis to be inhibited, DNA and RNA synthesis to be disrupted, ribosomes to be denatured, and protein formation to be inhibited; and (9) production of reactive oxygen species (ROS) [54]. The efficiency of silver nanoparticles is determined by factors such as particle size, shape, exposure period, compound type, and target. However, the size of silver is very important because the smaller diameter provides a larger surface area and hence superior antibacterial efficacy. The amount of silver in the solution must be high enough to inhibit the growth of bacteria [55]. The AgNPs get adsorbed on the surface of the Gram-negative bacterial cell wall resulting in breakdown of the cell wall as the Ag^+^ ions released from the AgNPs exhibit a strong antibacterial action [56,57]. 

## 5. Conclusions

In this study, the biocontrol agent *T. harzianum* was utilized as a reducing and stabilizing agent, resulting in the successful production of silver nanoparticles using an eco-friendly, affordable, and fast approach. UV-visible spectroscopy, DLS, zeta potential, TEM, SEM, and EDX were used to confirm the synthesis and NP structure, and to examine features such as size distribution, zeta potential, and morphology. Furthermore, in vitro and greenhouse antifungal activity against soil-borne pathogens *R. solani* (RS9), *F. fujikuroi* (FF10), and *M. phaseolina* (MP4) was demonstrated. AgNPs significantly inhibited hyphal development in the three fungal pathogens. *T. harzianum* isolates have been demonstrated to be capable of synthesizing a wide range of proteins and enzymes without the need for chemical reducers and stabilizers. Biosynthesized AgNPs have shown significant potential in protecting cotton plants from the fungal invasion caused by damping-off. In order to have more control over the size and polydispersity of AgNPs, more research is needed to discover the essential bioreducing and biotemplating molecules present in the hyphal extract. There is a need for more research on AgNPs in agroecosystems, and for more threat assessment research.

## Figures and Tables

**Table 1:** Fungicides and trichogenic AgNPs were used in the greenhouse to reduce damping-off of cotton seedlings.

Treatment	Rate of Treatments
1—autoclaved soil	*R. solani* (1 g), both of *F. fujikuroi* and *M. phaseolina* (50 g) for sterilized sorghum/kg soil
2—infested soil	*R. solani* (1 g), both of *F. fujikuroi* and *M. phaseolina* (50 g) for sterilized sorghum/kg soil
3—Moncut	2 g/kg seeds
4—Maxim XL	2 mL/L
5—AgNPs	100 µg mL^−1^
6—AgNPs	200 µg mL^−1^

**Table 2:** Analysis of variance of the antifungal effects of different concentrations of AgNPs and against the radial growth of *Fusarium fujikuroi* (FF10), *R. solani* (RS9), and *M. phaseolina* ( MP4).

Growth Variables and Sources of Variation	D.F	Mean Square	F. Value	*p* ≥ F
Concentration of AgNPs(C)	3	4.606	79.302	0.000
Fungus(F)	2	48.113	828.388	0.000
F × C	6	5.622	96.798	0.000
Error	22	0.058		

**Table 3:** Antifungal effects of different concentrations of silver NPs on the radial growth of *Fusarium fujikuroi* (FF10), *R. solani* (RS9), and *M. phaseolina* (MP4).

Radial Growth (cm)				
Treatment	FF10	RS9	MP4	Mean
AgNPs 20 μg/mL	6.000	9.000	6.750	7.250
AgNPs 40 μg/mL	5.333	9.000	6.333	6.889
AgNPs 100 μg/mL	4.000	2.250	4.167	3.472
Control	9.000	9.000	9.000	9.000
Mean	6.083	7.313	6.563	6.653

LSD of fungus × concentration = 0.393.

**Table 4:** ANOVA effects of fungus, treatments, and their interactions on several growth factors of Giza 90 cotton seedlings cultivated in contaminated soil under greenhouse conditions.

Growth Variables and Sources of Variation	D.F	Mean Square	F. Value	*p* ≥ F
Survival				
Fungi (F)	2	62.93	0.28	0.76
Treatments (T)	5	3805.20	16.89	0.00
F × T	10	71.46	0.32	0.97
Error	34	225.32		
Plant height				
Fungi (F)	2	21.65	1.72	0.19
Treatments (T)	5	212.09	16.84	0.00
F × T	10	52.94	4.20	0.00
Error	34	12.60		
Dry weight				
Fungi (F)	2	0.75	3.51	0.04
Treatments (T)	5	3.94	18.32	0.00
F × T	10	0.34	1.59	0.15
Error	34	0.22		

**Table 5:** The effect of a pathogenic fungus, treatments, and their interactions on the survival percentage of Giza90 cotton seedlings cultivated on infected soil in a greenhouse.

Treatments	Silver/Survival/Giza90			
	FF10 (%)	RS9 (%)	MP4 (%)	Mean (%)
AgNPs (100 μg/mL)	60.000	70.000	76.667	68.889
AgNPs (200 μg/mL)	90.000	86.667	93.333	90.000
Maxim ×L (2 mL)	86.667	86.667	90.000	87.778
Moncut (2 g)	86.667	83.333	83.333	84.444
Infested soil	30.000	16.667	26.667	24.445
Autoclaved soil	90.000	96.667	93.333	93.333
Mean	73.889	73.333	77.222	74.815

Before calculating the analysis of variance, the percentage values were converted into arcsine angles to achieve nearly constant variance. LSD (*p* ≤ 0.05) (transformed data) for treatments was 14.01. The LSD (*p* ≤ 0.05) for fungus is non-significant.

**Table 6:** ANOVA of the effects of specific fungi, treatments, and their interactions on some growth factors of Giza 94 cotton seedlings grown in a greenhouse on infected soil.

Growth Variables and Sources of Variation	D.F	Mean Square	F. Value	*p* ≥ F
Survival				
Fungi (F)	2	93.24	0.37	0.69
Treatments (T)	5	4579.66	18.25	0.00
F × T	10	408.13	1.63	0.14
Error	34	250.94		
Plant height				
Fungi (F)	2	7.89	0.24	0.79
Treatments (T)	5	272.93	8.17	0.00
F × T	10	40.05	1.20	0.33
Error	34	33.39		
Dry weight				
Fungi (F)	2	0.19	0.56	0.58
Treatments (T)	5	5.38	15.71	0.00
F × T	10	0.35	1.00	0.46
Error	34	0.34		

**Table 7:** The effects of various fungi, treatments, and their interactions on the survival percentage of Giza94 cotton seedlings grown in a greenhouse on infected soil.

Silver/Survival/Giza94								
Treatment	FF10 %Transformed ^a^		RS9 %Transformed ^a^		MP4 %Transformed ^a^		Mean %Transformed ^a^	
AgNPs (100 μg/mL)	40.000	38.853	16.667	15.000	80.000	63.930	45.556	39.261
AgNPs (200 μg/mL)	90.000	75.000	83.333	70.763	93.333	77.707	88.889	74.490
Maxim XL (2 mL)	80.000	67.860	76.667	65.840	76.667	61.910	77.778	65.203
Moncut (2 g)	83.333	70.077	93.333	77.707	90.000	75.000	88.889	74.261
Infested soil	16.667	19.223	30.000	28.077	6.667	12.293	17.778	19.864
Autoclaved soil	86.667	72.783	86.667	72.293	83.333	66.147	85.556	70.408
Mean	66.111	57.299	64.444	54.947	71.667	59.498	67.407	57.248

^a^ Before calculating the analysis of variance, the percentage values were converted into arcsine angles to achieve nearly constant variance. LSD (*p* ≤ 0.05) (transformed data) for treatments was 14.79. The LSD (*p* ≤ 0.05) for fungus was non-significant.

## Data Availability

Not applicable.
