# Correction: Zaki et al. Trichogenic Silver-Based Nanoparticles for Suppression of Fungi Involved in Damping-Off of Cotton Seedlings. *Microorganisms* 2022, *10*, 344

**DOI:** 10.3390/microorganisms13020383

**Published:** 2025-02-10

**Authors:** Shimaa A. Zaki, Salama A. Ouf, Kamel A. Abd-Elsalam, Amal A. Asran, Mohamed M. Hassan, Anu Kalia, Fawziah M. Albarakaty

**Affiliations:** 1 Botany and Microbiology Department, Faculty of Science, Cairo University, Giza 12613, Egypt; shim.shimshim@yahoo.com (S.A.Z.); salama@sci.cu.edu.eg (S.A.O.); 2 Plant Pathology Research Institute, Agricultural Research Centre, Giza 12619, Egypt; kamelabdelsalam@gmail.com (K.A.A.-E.); asran.amal@gmail.com (A.A.A.); 3 Department of Biology, College of Science, Taif University, Taif 21944, Saudi Arabia; m.khyate@tu.edu.sa; 4 Electron Microscopy and Nanoscience Laboratory, Department of Soil Science, College of Agriculture, Punjab Agricultural University, Ludhiana 141004, Punjab, India; kaliaanu@pau.edu; 5 Department of Biology, Faculty of Applied Science, Umm Al-Qura University, Makkah Al Mukarramah 21955, Saudi Arabia

## Error in Figure

In the original publication [1], there was a mistake in Figure 12 as published. Two images of the plants in the figure were identical; however, they were supposed to be different plants. The corrected Figure 12 and caption appear below.

Additionally, the units of “AgNPs” should be µg/mL rather than g/mL in the whole paper.

The authors state that the scientific conclusions are unaffected. This correction was approved by the Academic Editor. The original publication has also been updated.

## Figures and Tables

**Figure 12:**
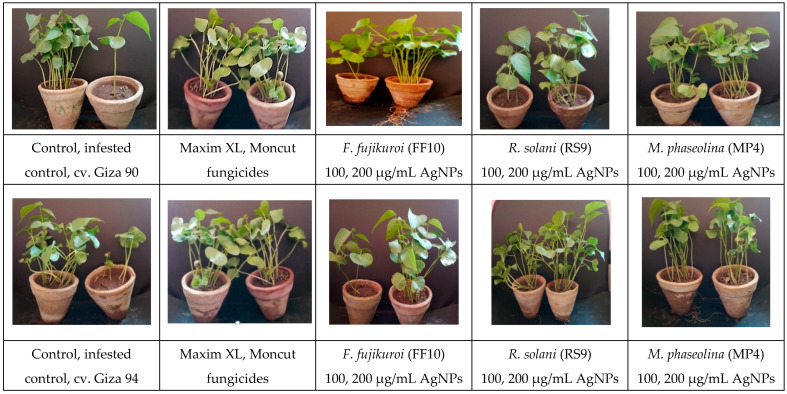
Uncoated cotton seeds sown in sterilized soil infested with three fungal pathogens, namely, *F. fujikuroi, R. solani*, and *M. phaseolina* as a negative control, uncoated cotton seeds sowed in sterilized soil as a positive control, treated with two fungicides (Maxim XL and Moncut) sowed in infested soil and coated with AgNPs (100, 200 µg/mL) in infested soil. The photo was obtained after 45 days of typical growth in a greenhouse.

## References

[1] Zaki S.A., Ouf S.A., Abd-Elsalam K.A., Asran A.A., Hassan M.M., Kalia A., Albarakaty F.M. (2022). Trichogenic Silver-Based Nanoparticles for Suppression of Fungi Involved in Damping-Off of Cotton Seedlings. Microorganisms.

